# Accumulation of bioactive metabolites in cultivated medical *Cannabis*

**DOI:** 10.1371/journal.pone.0201119

**Published:** 2018-07-23

**Authors:** Richard D. Richins, Laura Rodriguez-Uribe, Kiah Lowe, Rebekah Ferral, Mary A. O’Connell

**Affiliations:** 1 Department of Plant and Environmental Sciences, New Mexico State University, Las Cruces, New Mexico, United States of America; 2 Rio Grande Analytics, Las Cruces, New Mexico, United States of America; Michigan State University, UNITED STATES

## Abstract

There has been an increased use of medical *Cannabis* in the United States of America as more states legalize its use. Complete chemical analyses of this material can vary considerably between producers and is often not fully provided to consumers. As phytochemists in a state with legal medical *Cannabis* we sought to characterize the accumulation of phytochemicals in material grown by licensed commercial producers. We report the development of a simple extraction and analysis method, amenable to use by commercial laboratories for the detection and quantification of both cannabinoids and terpenoids. Through analysis of developing flowers on plants, we can identify sources of variability of floral metabolites due to flower maturity and position on the plant. The terpenoid composition varied by accession and was used to cluster cannabis strains into specific types. Inclusion of terpenoids with cannabinoids in the analysis of medical cannabis should be encouraged, as both of these classes of compounds could play a role in the beneficial medical effects of different cannabis strains.

## Introduction

Cannabinoids are the class of terpenophenolic secondary metabolites commonly produced by members of the *Cannabis* genus [1]. Within this class are specific compounds with notable bioactive effects; these effects often requiring post-biosynthetic processing, *i*.*e*. heat, to generate the more bioactive form [2]. Multiple peoples around the world have used the fibers, oils, resins, dried inflorescences and leaves of species within *Cannabis* for thousands of years [3]. There is some discussion of the number of species in the genus cultivated for use as marijuana, with the most recent assessment supporting one highly variable species, *C*. *sativa*, with variants, eg. var. *indica* as well as a number of strains within that species [4–6]. Strains have been selected for the production of hemp fiber or for the production of cannabinoid-containing resin. These two selection paths have isolated strains into types with and without the biosynthetic capability for the production of the psychoactive cannabinoid precursor Δ^9^-tetrahydrocannabinolic acid (THCA) [7]. *Cannabis* plants containing this compound are called marijuana, *Cannabis* plants that do not accumulate THCA to an appreciable level, (< 0.3%) are called hemp.

The *Cannabis* strains used for the production of cannabinoids are dioecious, with the maximal accumulation of these compounds accumulating on the unfertilized female inflorescence. The synthesis and accumulation of the cannabinoids occurs in trichomes on the surfaces of both leaves and inflorescences [8]. These plants are photoperiodic and commercial greenhouse production often utilizes clonally propagated material from female “mother plants” [9]. Cuttings are rooted and grown under vegetative light conditions (18 h or longer of light) for several weeks before the induction to flower. A switch to short day photoperiod, typically 12 h light, initiates floral development. Plants are then maintained for another 9 to 11 weeks and then the entire plant may be harvested and dried.

**Fig 1:**
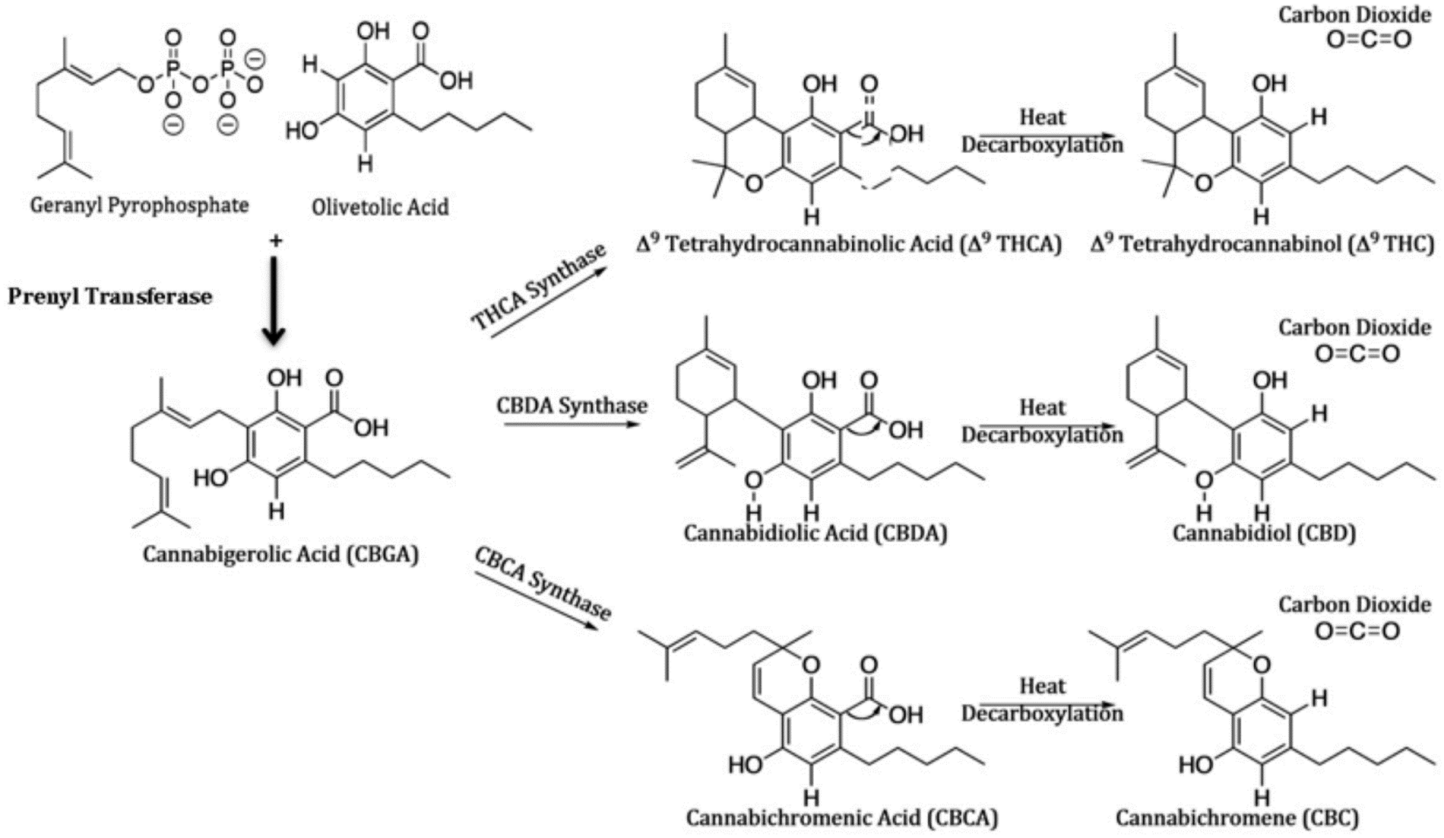
Biosynthetic schema for cannabinoids. This schema is derived from pathways reviewed in [2].

The biosynthetic pathway of the most abundant members of the cannabinoid class has been determined (reviewed in [2]). A schema of this pathway is presented in Fig 1. The enzymes and many genes for the pathway have been isolated and characterized in detail (reviewed in [10]). This includes the novel polyketide cyclase for the synthesis of olivetolic acid [11, 12], and the prenyl transferase, also called cannabigerolic acid synthase, that produces the central precursor cannabigerolic acid (CBGA) [13]. The specific synthases for the production of THCA [14], cannabidolic acid (CBDA) [15] and cannabichromenic acid (CBCA) [16] have also been characterized. Based on inheritance of chemical phenotypes, the genes for THCA synthase (THCAS) and CBDA synthase (CBDAS) are considered co-dominant alleles [7, 17]; while the gene for CBCA synthase is an independent locus. However, based on DNA sequencing and analysis of the transcripts expressed in marijuana and hemp samples, multiple linked loci for the THCAS and CBDAS genes are proposed [18, 19]. There are published genomes for *Cannabis*, and the genetic diversity for the biosynthetic pathway of cannabinoids in both marijuana and hemp strains is under investigation [18, 20].

The bioactive forms of the cannabinoids are produced following a light or heat induced decarboxylation reaction to generate cannabigerol (CBG), Δ^9^-tetrahydrocannabinol (Δ9-THC), cannabidiol (CBD) or cannabichromene (CBC). Other cannabinoids detected in plant samples include a further oxidation product of Δ9-THC, cannabinol (CBN) and cannabinoids produced from condensation of geranyl diphosphate with a variant of olivetolic acid, divarinic acid which has a propyl side chain instead of the pentyl side chain and results in Δ^9^-tetrahydrocannabivarin (Δ9-THCV). Based on the diversity of cannabinoid content, five unique chemotypes have been described [21–24]: chemotype I includes strains with primarily Δ9-THCV, chemotype II includes strains with similar levels of CBD and Δ9-THCV, chemotype III includes the hemp strains with virtually no Δ9-THCV and primarily CBD; chemotypes IV and V are less frequent and include strains with no Δ9-THCV and primarily CBG and strains with no cannabinoids.

In addition to the cannabinoids, medicinal *Cannabis* samples are also rich in bioactive terpenoids [25]; there appears to be a positive correlation between the accumulation of each of these classes of compounds [1]. The complexity of the terpenoid and cannabinoid composition in these samples should be routinely quantified to accurately and adequately determine the medicinal potential of a particular sample [26]. Inclusion of the terpenoid composition in a characterization of a medicinal marijuana sample also provides information about the provenance of the sample [1], as unique chemical abundances of specific terpenoids are predicted to be associated with chemotypes and species level taxa [5, 27]. As the aroma of the plant is largely the result of the terpenoid composition [25], human selections for this plant may have been based in part on the monoterpenoid and sesquiterpenoid based aroma and on the cannabinoid psychoactivity.

Within the past several decades, significant scientific evidence for the medical utility of both the floral material and specific cannabinoids from these plants has been presented for a number of human health diseases and conditions [9, 28–30]. In the United States of America, as of 2018, more than half of the states license the use of medical marijuana http://www.ncsl.org/research/health/state-medical-marijuana-laws.aspx; while the U.S. federal government continues to classify marijuana as a Schedule 1 drug https://www.justice.gov/iso/opa/resources/3052013829132756857467.pdf. As a result, there is very limited reliable data on the chemical composition of *Cannabis* strains, the variability within the plant for the production of these compounds and the interaction of the environment of production with the chemical quality of a specific strain. Much of the material currently available for medical use was selected from the numerous strains available from the illicit drug growers; understandably, the provenance and genetic backgrounds of these strains are not easily traceable.

Our past experience characterizing the sources of variability for secondary metabolite accumulation in medicinal plants like lavender [31] yerba mansa [32] or in *Capsicum* cultivars (chile) [33] lead us to investigate the variability for cannabinoid accumulation in medical marijuana produced by licensed growers in New Mexico.

There were several aims for the experiments reported here. We wanted to develop a rapid, reliable and inexpensive method to quantify the important bioactive compounds in medical marijuana, cannabinoids and terpenoids. We wanted to demonstrate the utility of the method using commercial material, available as medical marijuana in New Mexico. These studies were also designed to determine the consistency of total and relative cannabinoid and terpenoid accumulation within a given accession, and to see if leaf levels of cannabinoids could be used to predict eventual floral levels. To answer these questions, the concentration of five cannabinoids, Δ9-THC, CBD, CBG, Δ9-THCV, and CBC were determined in leaf and floral samples from 28 strains, collected at multiple times throughout the year. The terpenoid contents of selected medicinal strains are also presented. We also report here a reliable and sustainable gas chromatography method for the separation and quantification of these five neutral cannabinoids. This method has superior limit of detection (LoD) and limit of quantitation (LoQ) capabilities for these metabolites over the more common liquid chromatographic separations [34]. We present this method as a way to easily monitor both the cannabinoid and terpenoid compositions of medical marijuana plant material https://www.protocols.io/view/cannabinoid-and-terpenoid-extraction-and-analysis-rd4d28w.

## Materials and methods

### Plant material

All of the plant material characterized in this report will be referred to as *Cannabis*, without a species level description. The precise provenance of the plant material is difficult to trace, strain names are not protected, and growers’ descriptions of the plant material as “indica dominant” or “sativa dominant” do not necessarily reflect results obtained with genotyping [4].

For the developmental analyses, plants were grown in a commercial greenhouse in central New Mexico, under a medical marijuana producer license from the New Mexico Department of Health. Cuttings were propagated in Coco Air Max for 6 to 8 weeks with a 20 h light photoperiod. Plants were watered daily and were fertilized with Veg A&B and/or Bud A&B (Heavy 16, Signal Hill, CA) at a rate of between 500 and 1500 mg/L depending on the growth stage of the plants. Natural light was supplemented with artificial lighting using 600 watt CFL (compact fluorescent) and/or 1000-watt HPS (high-pressure sodium) lamps. The greenhouse temperature was set to 22°C; actual temperatures varied somewhat from a low of 17°C to a high of 33°C. Relative humidity varied between 35 and 65%. Plants were then transferred into 38 L pots and moved to a 12 h photoperiod to induce flowering. Again, natural sunlight was supplemented with artificial lighting as needed. Leaf and flower samples were collected at the days indicated post floral induction. In some cases, samples were collected from the top (upper third of plant) or bottom (lowest third of plant). For RNA isolation, leaf or flower samples were collected at “early” (4–5 weeks post floral induction), “mid” (6–7 weeks post floral induction, or late (8–11 weeks post floral induction). All of these samples were dried, but not “cured” prior to chemical extraction for metabolites. Samples for RNA extraction were not dried but frozen in liquid nitrogen and stored frozen until extraction.

A second source of plant material includes cured flowers from medicinal marijuana produced by licensed growers throughout the state of New Mexico. This material reflects the medical marijuana commercially available, and represents a wide range of strains. Those samples were submitted by producers as ‘cured’ medical cannabis. Curing is the method used to properly age and dry cannabis flower prior to consumption. A cured flower will typically have a moisture content of between 3% and 10%. All of the plant material was processed at Rio Grande Analytics, a licensed medicinal *Cannabis* testing laboratory. If the analyses utilize ‘cured’ medical cannabis samples that is indicated in the figure or table legend.

### Cannabinoid extraction and analysis

Cured/dried flowers or dried leaves were homogenized in 20 mL of reagent-grade acetone using a Polytron Homogenizer (Kinematica). The homogenates were placed on an orbital shaker for 1 h. Samples were diluted in four parts of acetone containing 1 mg/mL chlorophenol and 2 mg/mL phenacetin (ISS1 and ISS2, respectively). The samples were then centrifuged briefly to remove insoluble matter and analyzed using a Varian model 3900 GC-FID with an Rxi-35 column (15 m x 0.25 mm). Ultrapure nitrogen was employed as the carrier gas (flow rate: 1 mL/min). The injection volume was 2.0 μL, split 10, injector temperature 250°C, FID temperature 300°C. The temperature gradient for the analysis started at 45°C (with a 1 min hold), and increased to 240° at 10°/min, then increased to 280°C at 4°/min, then finally increased to 290°C at 20°/min with a one-minute hold. This method proved adequate to separate six of the more abundant cannabinoids (Δ9-THC, CBD, CBC, CBG, Δ9-THCV and CBN) and 21 terpenoids (Table 1). Cannabinoid standards were obtained from Restek (Bellefonte, PA) and Sigma-Aldrich (St Louis, MO); terpenoid standards were obtained from Restek as well.

**Table 1:** Linear calibration parameters for quantification of terpenoids and cannabinoids.

Peak ID	RT^a^	Analyte	Slope	Intercept	R2^b^
ISS^c^-1	3.67	Chlorobenzene	n/a	n/a	n/a
T1	4.19	α-Pinene	713	593	0.9998
T2	4.55	Camphene	729	510	0.9998
T3	4.98	β-Pinene	737	336	0.9998
T4	5.11	β-Myrcene	699	138	0.9998
T5	5.40	Delta-3-Carene	792	319	0.9998
T6	5.55	α-Terpinene	728	116	0.9998
T7	5.71	d-Limonene	825	186	0.9998
T8	5.89	*cis-*Ocimene^d^	857	15	0.9997
T9	5.96	p-Cymene	743	-118	0.9996
T10	6.02	*trans*-Ocimene	Combined T8 and T10 to calculate		
T11	6.27	γ-Terpinene	765	81	0.9998
T12	6.72	Terpinolene	750	237	0.9998
T13	6.96	Linalool	679	-265	0.9996
T14	7.79	(-)-Isopulegol	681	-533	0.9995
T15	9.35	Geraniol	693	-575	0.9994
T16	11.07	β-Caryophyllene	789	-301	0.9996
T17	11.56	α-Humulene	814	-367	0.9997
T18	12.48	*cis*-Nerolidal^d^	752	-767	0.9993
T19	12.86	*trans*-Nerolidal	Combined T18 and T19 to calculate		
T20	13.54	(-)-Guaiol	787	-655	0.9994
T21	14.42	(-)-α-Bisabolol	777	-22	0.9993
ISS-2	16.12	Phenacetin	n/a	n/a	n/a
C1	21.14	THCV	662	4649	0.9998
C2	21.83	CBC	661	6232	0.9997
C3	21.96	CBD	640	5426	0.9997
C4	23.12	Δ9-THC	680	5147	0.9998
C5	23.51	CBG	660	5600	0.9997
C6	23.92	CBN	597	5061	0.9997

The regression equation parameters for each analyte standard were determined using at least 10 different concentrations between 0 and 520 ng for the terpenoids and between 0 and 3880 ng for the cannabinoids. These parameters predict the mass of the analyte from the GC-FID peak area (y = mx + b; where y is the peak area, m is the slope, x is the mass in ng, and b is the intercept on the y axis). Peak ID is the code for each of the 21 terpenoids (T1-T21) and six cannabinoids (C1-C6), for which a calibration curve was generated.

^a^Retention time, RT;

^b^Correlation coefficient;

^c^Internal Spiked Standards (ISS);

^d^Concentration determined from sum of two peaks with unique RTs; n/a, not applicable.

### RNA isolation and analysis

Flowers and leaves were harvested and immediately frozen in liquid nitrogen; RNA was isolated using methods described earlier [35]; the quality of the RNA was confirmed by formaldehyde agarose gel electrophoresis. The primers and conditions for qRT-PCR analysis were developed based on methods described earlier [36]. The primers specific to *Cannabis* mRNA for the THCAS, CBDAS and the prenyl transferase are listed in Table 2. Calibration curves using purified amplicons for each gene were generated to allow for quantification of transcripts with expression data represented as pg transcript per 200 ng total RNA. Triplicate independent RNA isolations were performed (biological replication) and each RNA sample was analyzed in triplicate for qRT-PCR (technical replication). qRT-PCR was carried out with the KAPA SYBR FAST One-Step qRT-PCR Kit from KAPA BIOSYSTEMS Boston, MA. PCR cycling conditions for CBDAS were: cDNA synthesis 42°C 10 min; RT heat inactivation 95°C 5 min; amplification 40 cycles: 95°C 20 sec, 58.3°C 20 sec, 72°C 20 sec. PCR cycling conditions for THCAS were: cDNA synthesis 42°C 10 min, RT heat inactivation 95 °C 5 min; amplification 40 cycles: 95°C 20 sec, 62°C 20 sec, 72°C 20 sec. PCR cycling conditions for prenyl transferase were: cDNA synthesis 42°C 10 min, RT heat inactivation 95 °C 5 min; amplification 40 cycles: 95°C 20 sec, 62.2°C 20 sec, 72°C 20 sec.

**Table 2:** Primers for qRT-PCR analysis.

Gene	Forward	Reverse
**THCAS**	CTCGTATACACTCAACACGACC	GTAGGACATACCCTCAGCATCATG
**CBDAS**	GAGGCTATGGACCATTGA	GGACAGCAACCAGTCTAA
**Prenyl transferase**	CTGAGCCTCCAGAATCTGATAATC	GCCTTGAACATCAGAGACCAAC

## Results and discussion

### Detection of terpenoids and cannabinoids in a single GC-FID run

Gas chromatography is a useful approach for quantifying terpenoids and the abundant cannabinoids; however, achieving base line separation of some of the minor cannabinoids, CBC and CBD, can be difficult. Therefore, GC conditions were developed to improve the separation of these analytes. All of the cannabinoids were readily detected at 2 ng, and the peak area for these compounds increased linearly over the range from 2 ng to 3880 ng. Our working range for terpenoid detection was between 0.8 ng to 520 ng. The retention times for 19 terpenoid and 6 cannabinoid standards are presented in Table 1. This table also provides the linear regression parameters for the conversion of GC-FID peak areas to mass quantities of each of these specific metabolites.

**Fig 2:**
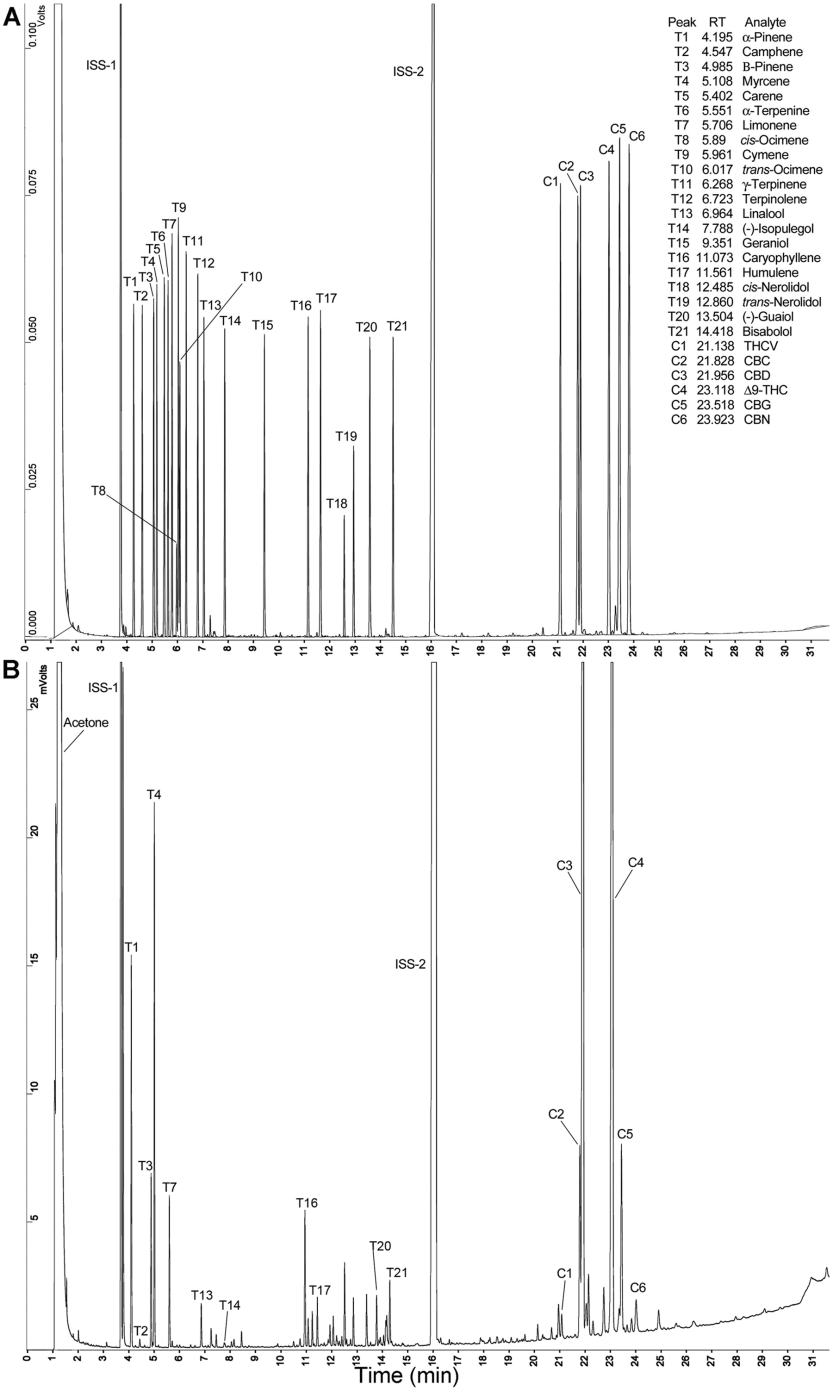
GC-FID separation of terpenoids and cannabinoids. Peaks are labeled with the Peak ID code. **A**. Mixture of 21 terpenoid and 6 cannabinoid chemical standards separated by GC-FID. **B**. Sample was extracted from C-Plus, a strain of *Cannabis* with approximately equal abundances of CBD (C3) and Δ9-THC (C4).

An example of the resolution of the terpenoids and cannabinoids in a cured *Cannabis* flower sample is presented in Fig 2. This method provides near baseline separation of the peaks for CBC (C2) and CBD (C3), even in this sample with a relatively high concentration of CBD. All six of the cannabinoids were detected in this strain, and we were able to identify and quantify a number of terpenoid peaks. This strain has relatively high levels of β-myrcene (T4), and α-pinene (T1).

The method described here for the analysis of cannabinoids and terpenoids differs from most published methods in that the cured plant samples were extracted with acetone. Most other published methods utilize methanol, methanol-acetonitrile, or methanol-chloroform as the extraction solvent [5, 26, 37]. Acetone was selected for this method because it is an excellent solvent for Δ9-THC. Acetone is additionally a less polar solvent, and therefore extracts fewer sugars and polysaccharides than does methanol. These sugars/polysaccharides build up in a GC column over time, which eventually compromises the performance of the column. Acetone would also be predicted to be an appropriate solvent for terpene/terpenoid extraction. Finally, acetone extracts of terpenes allowed for the detection and quantification of small monoterpenes such as α-pinene whereas methanol was retained longer by the RTx-35 GC column and interfered with the detection of these small terpenes.

A second difference in this assay for quantification of terpenoids and cannabinoids was the use of a single chromatographic system, GC-FID. Other groups have used a GC-MS and HPLC system to characterize both of these classes of metabolites in medical cannabis samples [26], or two different GC runs [5, 27]. The use of nitrogen as the carrier gas was a key element in the method described here. Helium gas, which is the more common carrier gas in GC methods for cannabinoids has become difficult and expensive to obtain. Nitrogen gas is readily and sustainably available; further the switch to nitrogen gas improved the separation of the CBC and CBD compounds on the chromatogram. These differences allow this method to recover and detect the lower abundance cannabinoids. One group has quantified terpenoids and cannabinoids using a single GC-FID run [1]; they also used nitrogen as a carrier gas. This group extracted their plant samples sequentially three times with ethanol for a total of 100 mL for a 1 g plant sample. In contrast we use only one acetone extraction, 1 g in 20 mL, homogenized and then shaken on an orbital shaker 1 h. This much simpler protocol recovers equivalent levels of terpenoids and cannabinoids as demonstrated below. There is a limitation to this method; we cannot detect the free acid forms of the cannabinoids, only the neutral forms of the cannabinoids. The heat in the protocol converts all free acid forms to neutral forms. In order to quantify the free acid forms of cannabinoids, the samples must be derivatized prior to GC separation [37].

### Comparison of cannabinoid content in leaf and floral samples from medicinal marijuana plants

The cannabinoids present in leaf and floral samples of 16 different medicinal marijuana plants were determined. The levels of six different cannabinoids in floral samples are listed in Table 3 and the levels in leaf samples are listed in Table 4. In both tables the levels are reported as percent of dry weight of the respective organ, these organs were collected from plants at 50 to 65 d post-light induction. As expected the levels of cannabinoids are variable between strains, and the levels in floral tissues are much higher than in leaf tissues. Δ9-THC levels in floral tissues range from 21% in strain Holy Power, to 3% in Juanita. Δ9-THC levels in leaf tissues were generally at least ten fold lower and range from 2.7% in strain Crystal Cookies to 0.3% in Love Lace.

**Table 3:** Inflorescence cannabinoid content of *Cannabis* plants cultivated in a greenhouse.

Strains	Δ9-THC	CBD	CBC	CBG	Δ9-THCV
Holy Power	21.53 ± 2.04	0.09 ± 0.02	0.34 ± 0.02	0.62 ± 0.06	0.16 ± 0.01
Green Crack	20.30 ± 2.33	0.10 ± 0.01	0.44 ± 0.05	1.19 ± 0.14	0.16 ± 0.01
Platinum Scout	18.70 ± 0.26	0.00 ± 0.00	0.34 ± 0.02	0.40 ± 0.05	1.01 ± 0.03
Green Crack 2015	17.17 ± 1.33	0.07 ± 0.00	0.20 ± 0.03	1.51 ± 0.17	0.12 ± 0.01
Crystal Cookies	17.07 ± 1.85	0.08 ± 0.01	0.51 ± 0.07	0.70 ± 0.08	0.97 ± 0.12
Oracle	17.02 ± 0.40	0.07 ± 0.01	0.25 ± 0.01	0.67 ± 0.05	0.08 ± 0.01
Lavendar Jones 2015	16.53 ± 2.80	0.06 ± 0.01	0.32 ± 0.05	0.29 ± 0.04	0.10 ± 0.01
Lavendar Jones	16.27 ± 1.21	0.08 ± 0.01	0.50 ± 0.02	0.21 ± 0.04	0.09 ± 0.00
Walker Kush	16.07 ± 0.85	0.07 ± 0.02	0.28 ± 0.01	0.55 ± 0.06	0.08 ± 0.01
Platinum Gorilla	15.80 ± 0.60	0.06 ± 0.00	0.37 ± 0.02	0.28 ± 0.06	0.40 ± 0.02
White Widow	15.63 ± 0.91	0.07 ± 0.00	0.17 ± 0.03	0.19 ± 0.03	0.05 ± 0.01
FLO	15.30 ± 1.52	0.07 ± 0.01	0.21 ± 0.02	0.53 ± 0.09	0.07 ± 0.02
Grandaddy Purple	14.30 ± 0.79	0.06 ± 0.01	0.22 ± 0.01	0.32 ± 0.00	0.05 ± 0.00
Platinum Buffalo	13.90 ± 0.75	0.00 ± 0.01	0.33 ± 0.02	0.23 ± 0.02	1.30 ± 0.05
RX	12.73 ± 0.45	0.06 ± 0.00	0.16 ± 0.01	0.96 ± 0.03	0.22 ± 0.02
Twisted Velvet	12.47 ± 1.29	0.05 ± 0.01	0.35 ± 0.03	2.08 ± 0.17	0.13 ± 0.02
Double Royal Kush	11.97 ± 0.35	0.05 ± 0.01	0.27 ± 0.02	0.56 ± 0.09	0.08 ± 0.02
Lavendar	11.91 ± 3.91	0.04 ± 0.01	0.15 ± 0.02	0.53 ± 0.17	0.05 ± 0.01
Skywalker	11.74 ± 2.09	0.04 ± 0.00	0.14 ± 0.00	0.88 ± 0.09	0.07 ± 0.00
Blue Dream	10.99 ± 2.41	0.05 ± 0.01	0.13 ± 0.01	0.10 ± 0.02	0.03 ± 0.01
Purple Fat Pie	10.85 ± 1.07	0.07 ± 0.02	0.27 ± 0.02	0.33 ± 0.03	0.05 ± 0.01
Romulin	10.21 ± 2.55	0.05 ± 0.01	0.17 ± 0.01	0.43 ± 0.02	0.13 ± 0.03
Blue Cherry Pie	9.80 ± 2.25	0.06 ± 0.04	0.14 ± 0.07	0.09 ± 0.02	0.10 ± 0.06
Cold Creek Kush	8.88 ± 4.28	0.04 ± 0.00	0.14 ± 0.00	0.31 ± 0.10	0.06 ± 0.00
Alien Blues	8.66 ± 1.16	0.11 ± 0.03	0.33 ± 0.09	0.26 ± 0.06	0.09 ± 0.06
Thunderstruck	5.94 ± 0.63	9.84 ± 1.15	0.62 ± 0.06	0.20 ± 0.05	0.05 ± 0.01
Sour Willie	5.73 ± 2.26	0.03 ± 0.00	0.14 ± 0.04	0.05 ± 0.00	0.02 ± 0.00
Love Lace	4.20 ± 0.53	8.88 ± 1.10	0.56 ± 0.04	0.24 ± 0.01	0.39 ± 0.03
Bohdi Tree	3.83 ± 1.55	0.02 ± 0.00	0.05 ± 0.00	0.08 ± 0.00	0.02 ± 0.00
Juanita	3.03 ± 0.37	4.71 ± 0.81	0.21 ± 0.00	0.07 ± 0.00	0.02 ± 0.00

Concentrations expressed as % flower dry wt (avg ± std dev): Δ^9^tetrahydrocannabinol (**Δ9**-THC); cannabidiol (CBD); Δ^9^tetrahydrocannabivarin (**Δ9**-THCV); cannabigerol (CBG); and cannabichromene (CBC). Strains are listed in rank order high to low Δ9-THC content.

**Table 4:** Leaf cannabinoid content of *Cannabis* plants cultivated in a greenhouse.

Strains	Δ9-THC	CBD	CBC	CBG	Δ9-THCV
Crystal Cookies	2.69 ± 0.49	0.00 ± 0.00	0.19 ± 0.04	0.01 ± 0.00	0.10 ± 0.02
Platinum Gorilla	2.56 ± 0.46	0.12 ± 0.23	0.22 ± 0.03	0.01 ± 0.00	0.02 ± 0.01
Green Crack 2017	2.03 ± 0.50	0.01 ± 0.00	0.39 ± 0.11	0.02 ± 0.00	0.01 ± 0.01
Twisted Velvet	1.62± 0.43	0.00 ± 0.00	0.45 ± 0.05	0.02 ± 0.01	0.00 ± 0.01
Double Royal Kush	1.56± 0.76	0.01 ± 0.00	0.41 ± 0.29	0.02 ± 0.01	0.02 ± 0.01
Blue Dream	1.47± 0.37	0.01 ± 0.00	0.12 ± 0.03	0.01 ± 0.00	0.01 ± 0.00
Platinum Buffalo	1.38± 0.44	0.00 ± 0.00	0.69 ± 0.07	0.01 ± 0.01	0.01 ± 0.03
Platinum Scout	1.27± 0.27	0.00 ± 0.00	0.12 ± 0.12	0.00 ± 0.00	0.00 ± 0.00
Blue Cherry Pie	1.21± 0.65	0.00 ± 0.00	0.33 ± 0.16	0.00 ± 0.00	0.00 ± 0.00
White Widow	1.01± 0.17	0.01 ± 0.00	0.05 ± 0.00	0.00 ± 0.00	0.01 ± 0.00
Cold Creek Kush	1.01± 0.20	0.01 ± 0.00	0.17 ± 0.04	0.01 ± 0.00	0.01 ± 0.00
Walker Kush	1.00± 3.00	0.01 ± 0.00	0.18 ± 0.06	0.01 ± 0.01	0.01 ± 0.00
Lavendar	0.99± 0.28	0.01 ± 0.00	0.09 ± 0.04	0.01 ± 0.00	0.01 ± 0.00
Skywalker	0.94± 0.24	0.01 ± 0.00	0.14 ± 0.03	0.01 ± 0.00	0.01 ± 0.00
Grandaddy Purple	0.91± 0.17	0.01 ± 0.00	0.07 ± 0.01	0.01 ± 0.00	0.01 ± 0.00
Sour Willie	0.89 ± 0.22	0.01 ± 0.00	0.15 ± 0.06	0.00 ± 0.00	0.01 ± 0.00
Purple Fat Pie	0.85 ± 0.22	0.00 ± 0.00	0.04 ± 0.03	0.00 ± 0.00	0.00 ± 0.00
Holy Power	0.84 ± 0.56	0.00 ± 0.00	0.08 ± 0.05	0.01 ± 0.02	0.00 ± 0.00
Romulin	0.83 ± 0.13	0.01 ± 0.00	0.07 ± 0.01	0.01 ± 0.00	0.01 ± 0.00
Oracle	0.83 ± 0.19	0.01 ± 0.00	0.20 ± 0.12	0.01 ± 0.01	0.01 ± 0.00
Alien Blues	0.78 ± 0.38	0.01 ± 0.00	0.09 ± 0.01	0.02 ± 0.01	0.00 ± 0.00
RX	0.74 ± 0.07	0.01 ± 0.00	0.06 ± 0.00	0.01 ± 0.00	0.02 ± 0.00
SkyWalker	0.73 ± 0.19	0.01 ± 0.00	0.04 ± 0.01	0.02 ± 0.01	0.01 ± 0.00
FLO	0.67 ± 0.12	0.01 ± 0.00	0.10 ± 0.02	0.01 ± 0.00	0.01 ± 0.00
Thunderstruck	0.66 ± 0.15	1.24 ± 0.28	0.15 ± 0.02	0.04 ± 0.04	0.00 ± 0.00
Lavendar Jones 2015	0.58 ± 0.04	0.01 ± 0.00	0.09 ± 0.01	0.01 ± 0.00	0.01 ± 0.00
Green Crack 2015	0.58 ± 0.10	0.01 ± 0.00	0.05 ± 0.01	0.01 ± 0.00	0.01 ± 0.00
Bohdi Tree	0.55 ± 0.15	0.01 ± 0.00	0.03 ± 0.01	0.00 ± 0.00	0.01 ± 0.00
Lavendar Jones 2017	0.46 ± 0.09	0.00 ± 0.00	0.15 ± 0.04	0.00 ± 0.00	0.00 ± 0.00
Juanita	0.41 ± 0.15	0.70 ± 0.27	0.08 ± 0.04	0.00 ± 0.00	0.01 ± 0.00
Love Lace	0.26 ± 0.03	0.69 ± 0.02	0.09 ± 0.02	0.00 ± 0.00	0.00 ± 0.00

Concentrations expressed as % leaf dry wt (avg ± std dev): Δ^9^tetrahydrocannabinol (Δ9-THC); cannabidiol (CBD); Δ^9^tetrahydrocannabivarin (Δ9-THCV); cannabigerol (CBG); and cannabichromene (CBC). Strains are listed in rank order, high to low Δ9-THC content.

The other important medical cannabinoid CBD, was barely detectable in leaf or floral tissue of most of the strains except Alien Blues, Thunderstruck, Love Lace and Juanita. In three strains, Thunderstruck, Love Lace and Juanita, the CBD levels were higher than Δ9-THC and ranged between 9% and 4.7% CBD. A third cannabinoid, CBC, was present at similar levels in leaves and floral tissues, and varied between strains. Platinum Buffalo had the highest leaf levels, 0.69%, while Bohdi Tree again had the lowest 0.03%. Thunderstruck had the highest CBC levels in floral tissues, 0.62% while Bohdi Tree had the lowest floral levels at 0.05%. CBG the precursor for Δ9-THC, CBD or CBC was also readily detected in floral samples and at trace levels in leaf samples. CBG levels in floral samples ranged between 2.1% in Twisted Velvet to 0.05% in Sour Willie. The variant cannabinoid Δ9-THCV was also detected in floral samples and at much lower levels in leaf samples. The strain Platinum Buffalo had the highest levels in floral samples at 1.3%.

The higher levels of cannabinoids in floral versus leaf tissue is expected and has been described by many other investigators [2, 9, 22]. The levels of Δ9-THC and CBD among the marijuana plants cultivated in New Mexico for medical applications are similar to the levels reported for cannabinoids in medical marijuana produced in other states and countries [1, 26, 38]. Typical levels for “high Δ9-THC” (chemotype I) strains range from 15 to 22%, and levels for “equal CBD to THC” (chemotype II) strains range from 5 to 10% of each cannabinoid [22].

### Time course for accumulation of Δ9-THC in flowers of medicinal marijuana

**Fig 3:**
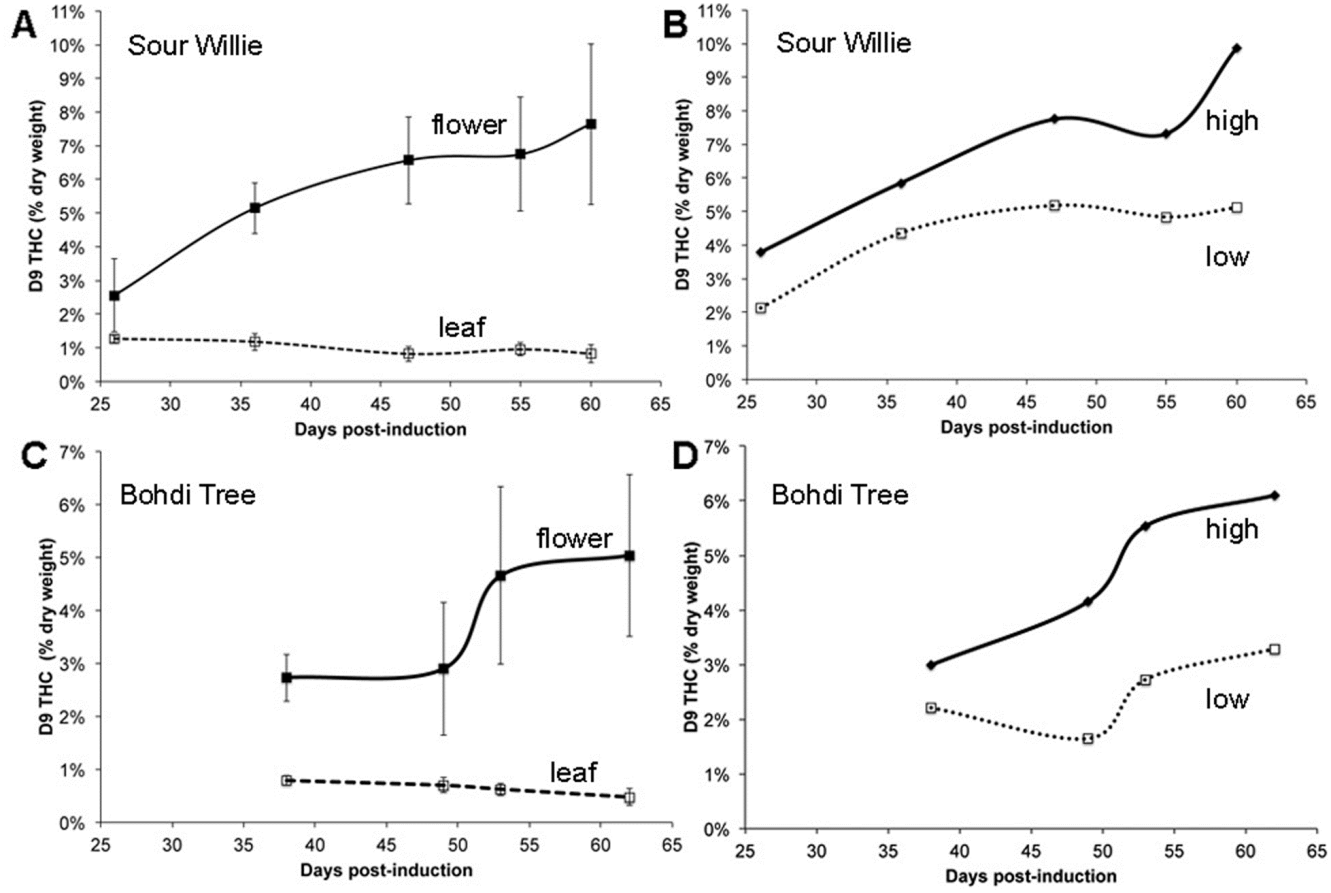
Accumulation of Δ9-THC in organs of Sour Willie or Bohdi Tree following floral induction. Δ9-THC levels in samples collected from Sour Willie (**A, B**) or Bohdi Tree (**C, D**) at days post-induction are represented. Panels A and C report average (n = 3) Δ9-THC levels in floral samples (solid line) and leaf samples (dotted line). Panels B and D report Δ9-THC levels in floral samples from the top of the plant (solid) or bottom of the plant (dotted line).

The induction of flowering in the clonally propagated plants is initiated by a shift to a short-day photoperiod. Floral and leaf samples were collected from two strains, Sour Willie and Bohdi Tree at multiple intervals following the floral induction. This plant material was collected from greenhouse grown plants. The accumulation of Δ9-THC in these samples was determined and is shown in Fig 3. As expected the Δ9-THC content of the samples increases in the floral samples with increasing time post-induction, this is observed in both strains. The levels of Δ9-THC in the leaf samples decrease slightly during this same time period. There is considerable variation in Δ9-THC content in the floral samples at each time point for collection. These are the averages of multiple flowers collected from different parts of the plant. When those flowers are separated into their locations on the plant, high, middle and low, a pattern of accumulation is detected. Floral samples from the top or high portion of the plant have higher levels of Δ9-THC than floral samples from the lower portion of the plant (Fig 3B and 3D). This was observed in both Sour Willie and Bohdi Tree and is anecdotal knowledge among producers of this crop. This same pattern of accumulation was observed in two additional strains, increasing the biological replication of this observation. Within each strain there is only a single data point for a floral position at a unique time, we see exactly the same pattern across four strains (S1 Fig); the Δ9-THC content is highest in flowers in the upper third, lowest in the flowers from the lower third, and intermediate in flowers from the middle third of the plant.

The observation that the cannabinoid content increases during floral development has been described by other investigators for plants cultivated in vitro as well as in greenhouse settings [22, 39]. The quantification of the variability within the plant for floral content of cannabinoids has not been described in the literature previously. The Δ9-THC content in flowers from the upper levels of the plant was close to twice as high as that in flowers from lower levels of the plant; this was observed in two distinct strains.

**Fig 4:**
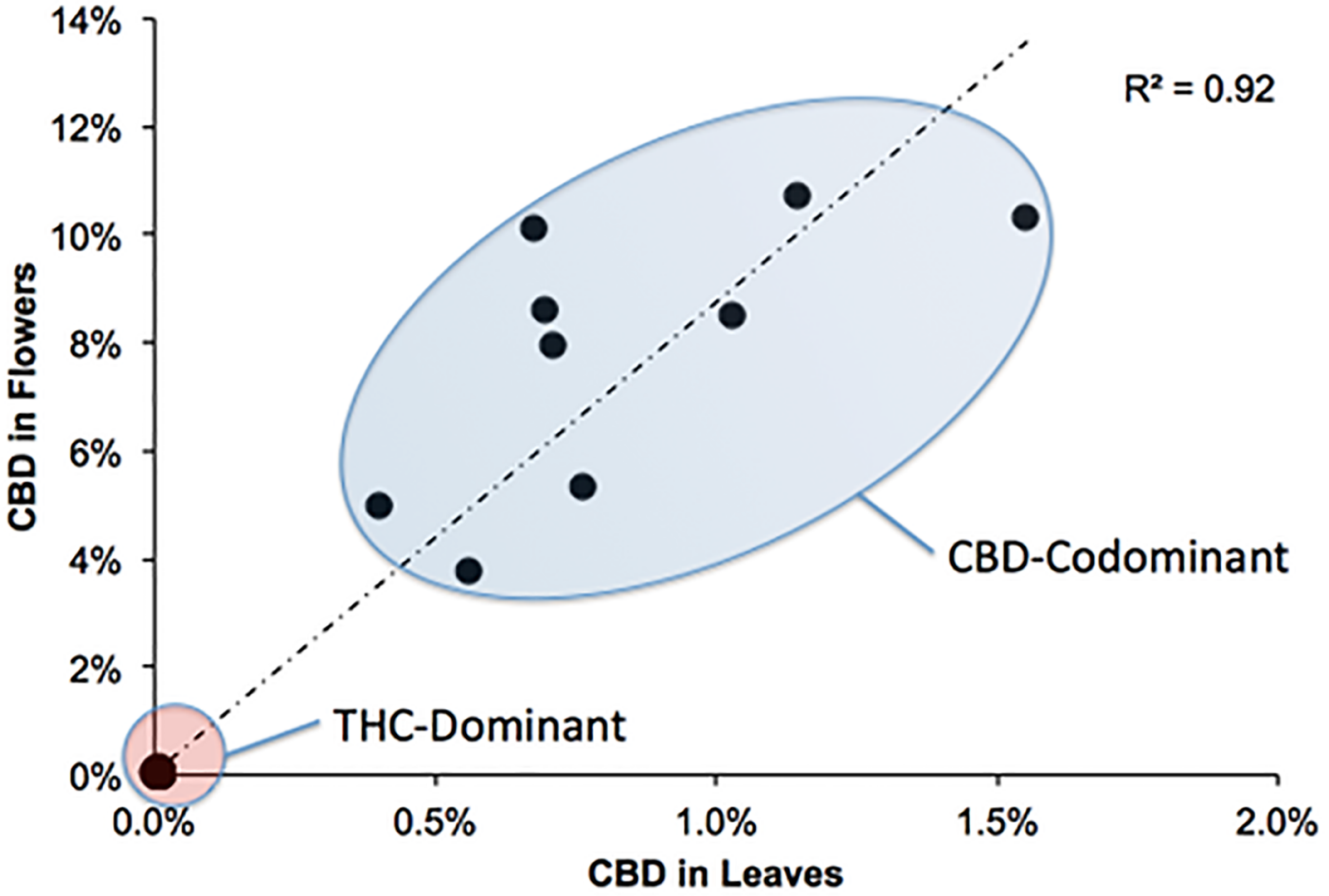
Paired comparisons of CBD levels in leaf and floral samples of *Cannabis* plants. Flowers and leaves were collected from 16 *Cannabis* strains. The Δ9-THC dominant strains fall within the red circle, while the CBD co-dominant strains fall within the blue circle.

Given the somewhat unusual sourcing of plants grown in medical marijuana facilities, the ability to predict whether a particular plant will produce flowers with high levels of CBD or Δ9-THC is desirable. For example, does the cannabinoid content of vegetative leaves, those on the plant prior to flower induction predict the cannabinoid content of the mature flowers that develop later on that plant. A comparison of the levels of CBD in vegetative leaves and mature flowers is plotted for samples from 16 strains (Fig 4). There is a positive correlation between the CBD content of leaf and flower samples, with an R^2^ value of 0.92. If a strain is going to have primarily Δ9-THC in its flowers, there will be virtually no CBD in its vegetative leaves. Conversely, if a strain is going to have appreciable levels of CBD in its flowers, then the vegetative leaves will have at least 0.5% CBD content. We also tested for a correlative relationship between abundance of Δ9-THC in leaves and flowers and detected a slight trend between Δ9-THC content in leaf and only the flowers from the upper portions of the plant however there was insufficient statistical support for this relationship (S1 Fig). Testing vegetative leaves to determine if a medical marijuana strain predicted to have high CBD floral content will provide valuable information early on in the management of the plant.

### Terpenoid content and composition in medical cannabis

**Fig 5:**
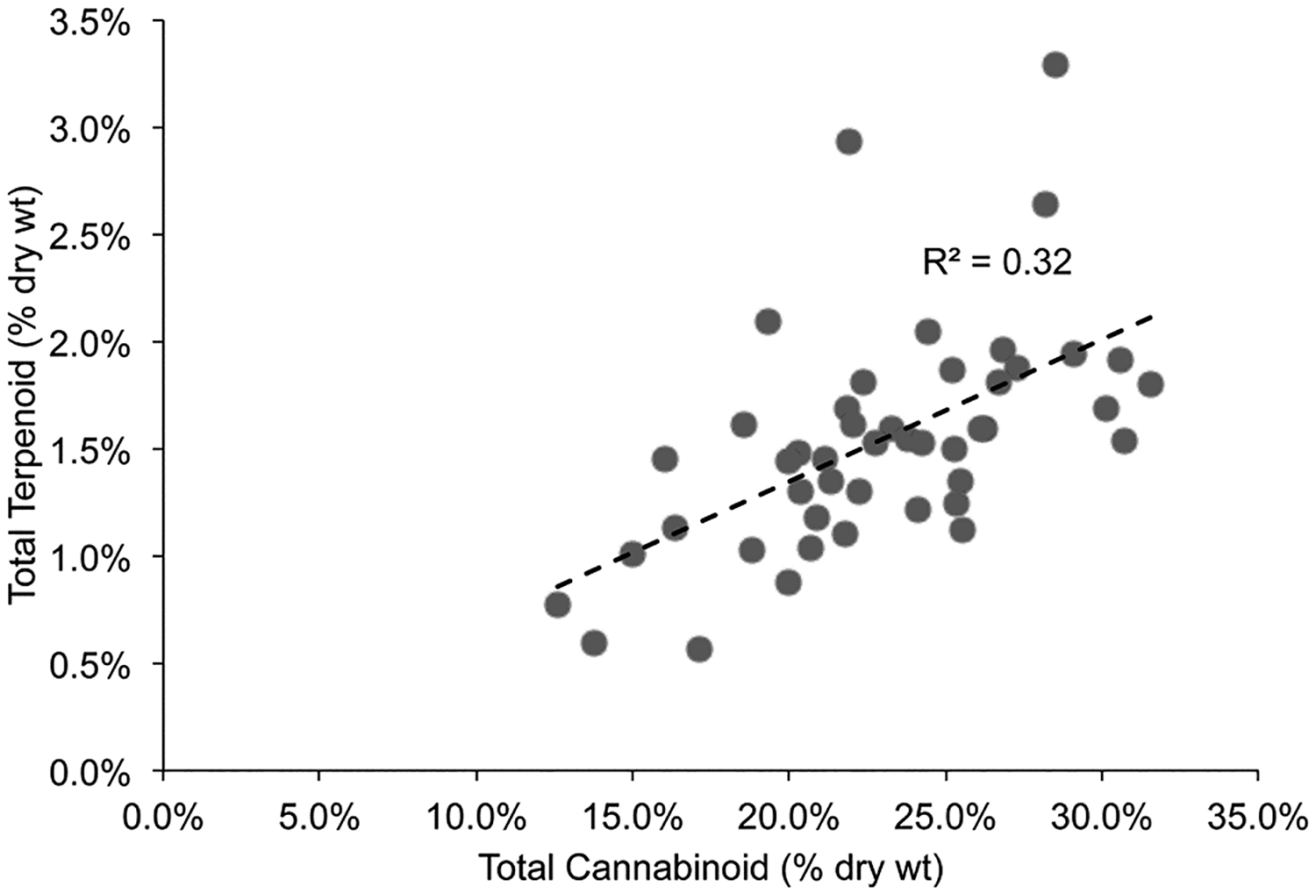
Paired comparisons of total cannabinoid and total terpenoid levels. Cured trimmed flowers were extracted and the terpenoid and cannabinoid composition determined by GC-FID. The total values for cannabinoids and terpenoids in each sample were plotted.

A detailed analysis of the terpenoid and cannabinoid content of commercially generated medical *Cannabis* floral samples was conducted. The aim of this analysis was to compare/contrast the terpenoid composition of these samples among the various strains. The growth conditions of the plant, the harvest of the samples and their curing process were all under the purview of the producer. The strain names for these samples are provided in the supporting information (S1 Table), but we have somewhat limited confidence in the provenance of the strain name. However, the chemical composition of this material used medicinally in New Mexico has been accurately determined. In the supplemental material we provide the abundances of 19 terpenoids and 6 cannabinoids determined on 72 strains of medical *Cannabis*. The totals of these values were plotted to look for correlations in levels (Fig 5).

The total terpenoid content ranged between 0.6 and 3.3%, while the total cannabinoid content ranged between 12.6–31.5% in these samples. We found a slight positive correlation between total cannabinoid content and total terpenoid content (Fig 5), with an R^2^ = 0.32. There are limited biosynthetic interactions between these two biosynthetic pathways, so a strong correlation based on shared biochemical pathways is not predicted. One group has reported a positive correlation between the terpenoid and cannabinoid content [1] of selected strains developed in the Netherlands for medicinal uses. Those authors do not consider the abundance of these metabolites to be linked metabolically either. The positive association between the abundance of terpenoids and cannabinoids in Fig 5 probably reflects increased production of all metabolites, including oils in larger healthier floral buds.

**Fig 6:**
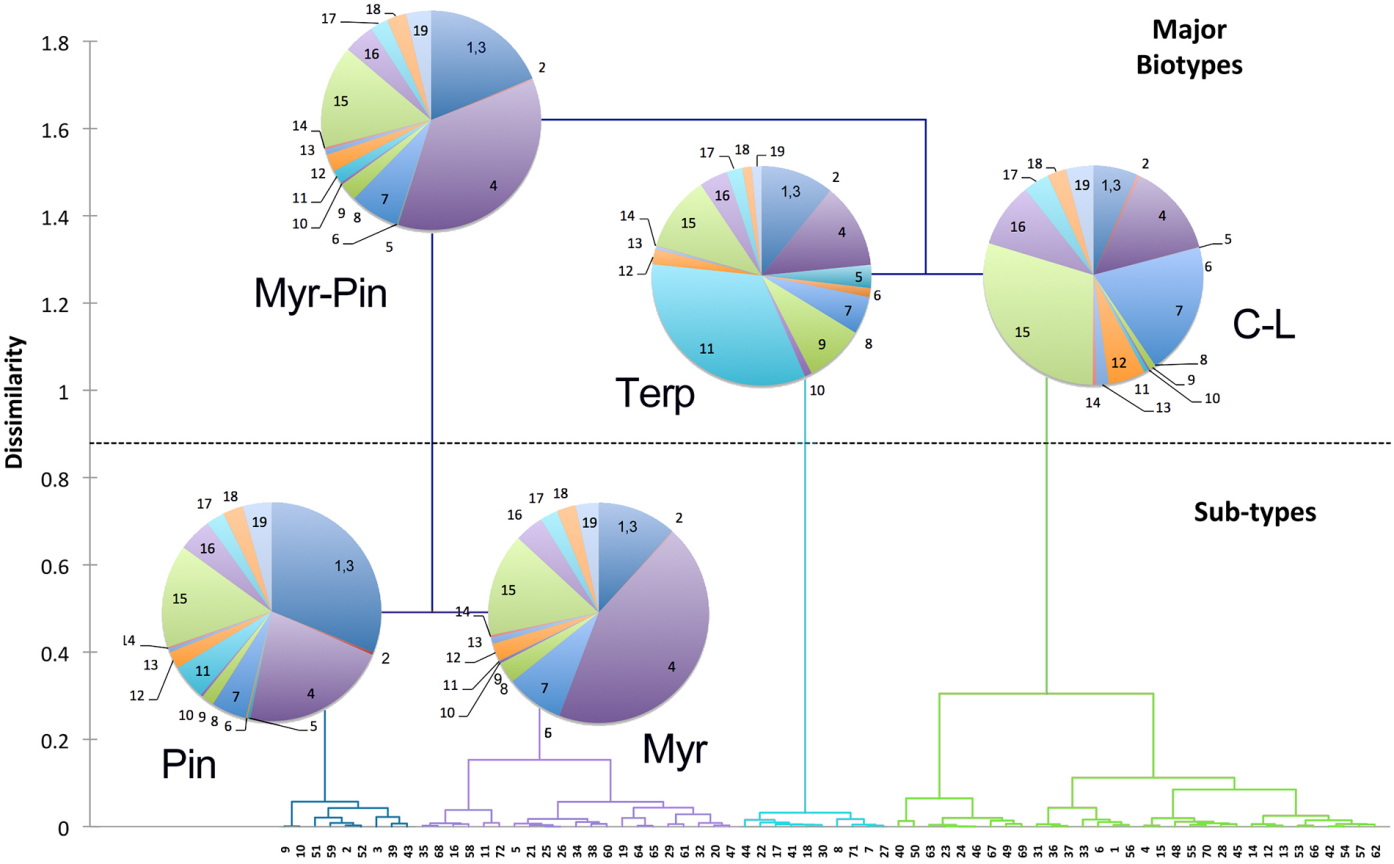
Hierarchical clustering of medical *Cannabis* strains based on floral terpenoid composition. The relative abundance of 19 terpenoids detected by GC-FID in cured floral samples from 72 medical *Cannabis* strains were used to generate the tree using the agglomerative hierarchical clustering method in the XLSTAT plug-in for Excel. Pie charts of a sample terpenoid composition for individuals within each clade are shown at the branch points, terpenoid composition is indicated by numerical code (Table 1, 1 = T1, 2 = T2, etc). Terpenoid types are identified: α- and β-pinene, Pin; β-myrcene, Myr; β-caryophyllene, C; d-limonene, L; terpinolene, Terp.

We also tested for chemotypes within the medical cannabis population based on terpenoid profiles. For that analysis we clustered the *Cannabis* strains using the relative abundance of 19 unique terpenoids in 72 different strains. The dendrogram that resulted from this analysis is presented in Fig 6. The specific composition and strain identifications are presented in supporting information (S1 Table). Pie charts for selected strains are presented along with the color code for the terpenoid composition in S1 Table. As demonstrated in Fig 6, there were three major clades or chemotypes: a type rich in β-myrcene and α- and β-pinenes (30 strains); a type rich in terpinolene (10 strains); and a type rich in d-limonene and β-caryophyllene (32 strains). We also noted two sub-types with reduced statistical support, an α- and β-pinene rich clade (9 strains); and a β-myrcene rich clade (21 strains). We did not test plants with high CBD/low Δ9-THC content and it is likely that additional terpenoid chemotypes will be found in those plants. The biosynthetic pathway for the monoterpenoids and sesquiterpenoids present in the *Cannabis* samples are highly interrelated and positive and negative correlations between the accumulations of specific compounds are expected.

Several other groups around the world have used terpenoid profiles to categorize *Cannabis* strains [1, 22, 26, 40]. In these cases the authors used a principal components analysis approach to identify metabolites important in either clustering or discriminating specific strains. Clustering analysis of medicinal *Cannabis* samples from Canada identified β-pinene, β-myrcene, d-limonene, and β-caryophyllene among other terpenoids as important discriminators [26]. PCA and hierarchical clustering of 11 medicinal *Cannabis* samples from the Netherlands also demonstrated the importance of terpenoids in the classification of *Cannabis* strains; again α- and β-pinene, β-myrcene, d-limonene, and β-caryophyllene were noted among other metabolites [1]. Recently, a PCA and hierarchical analysis of 30 cultivars from a medical *Cannabis* dispensary in California also identified five major groups based on the abundance of 16 terpenoids in these samples [40]. Our approach of agglomerative clustering and then inspection of pie chart displays of terpenoid composition, allowed us to identify the clades in the trees with their most abundant terpenoid.

### Transcriptional control of cannabinoid biosynthetic genes

**Fig 7:**
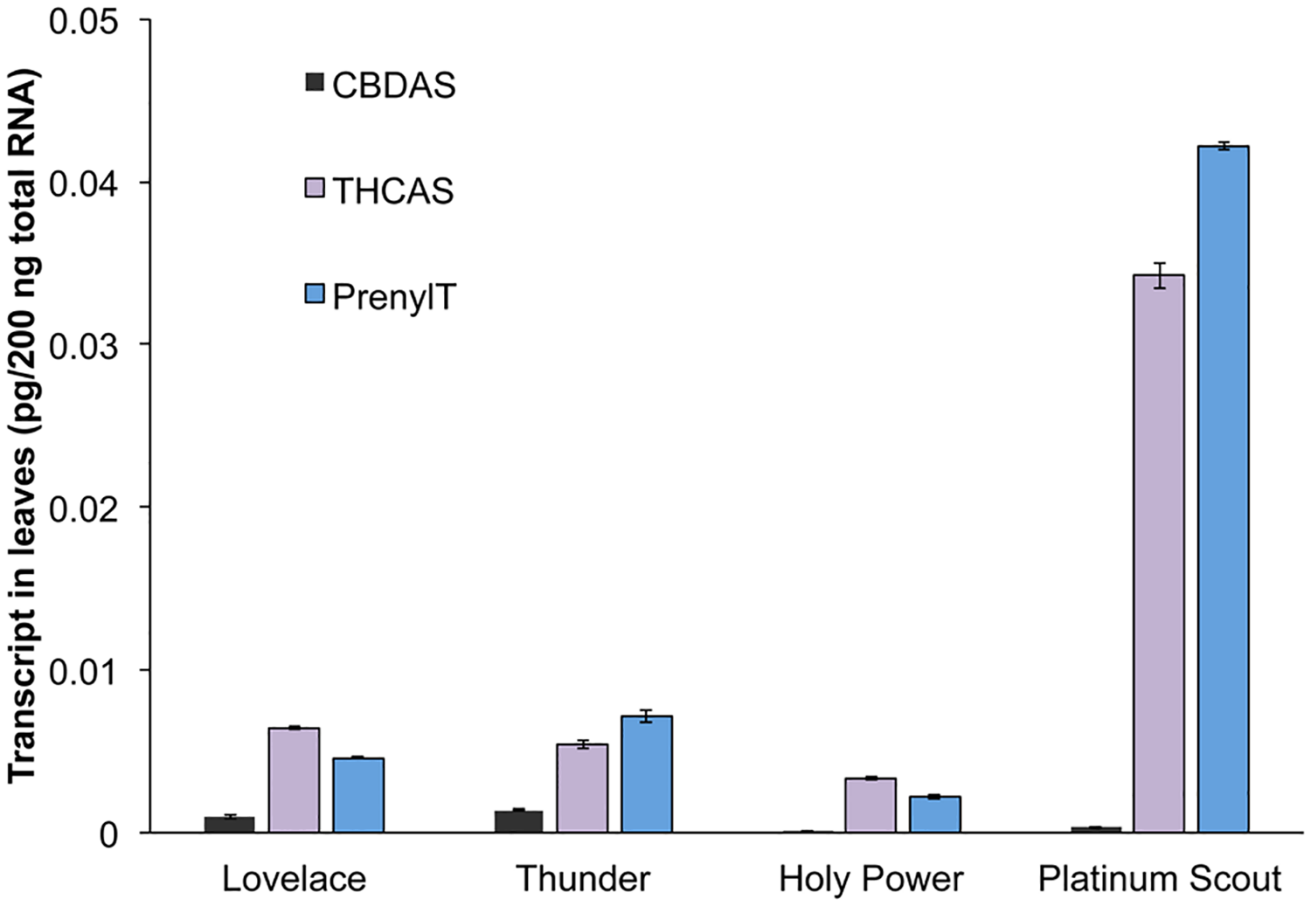
Transcript accumulation in leaf samples of cannabinoid biosynthetic genes. Leaf RNA from the indicated strains were assayed in triplicate by qRT-PCR, using primers for CBDAS (black), for THCAS (purple) or prenyl transferase (blue).

**Fig 8:**
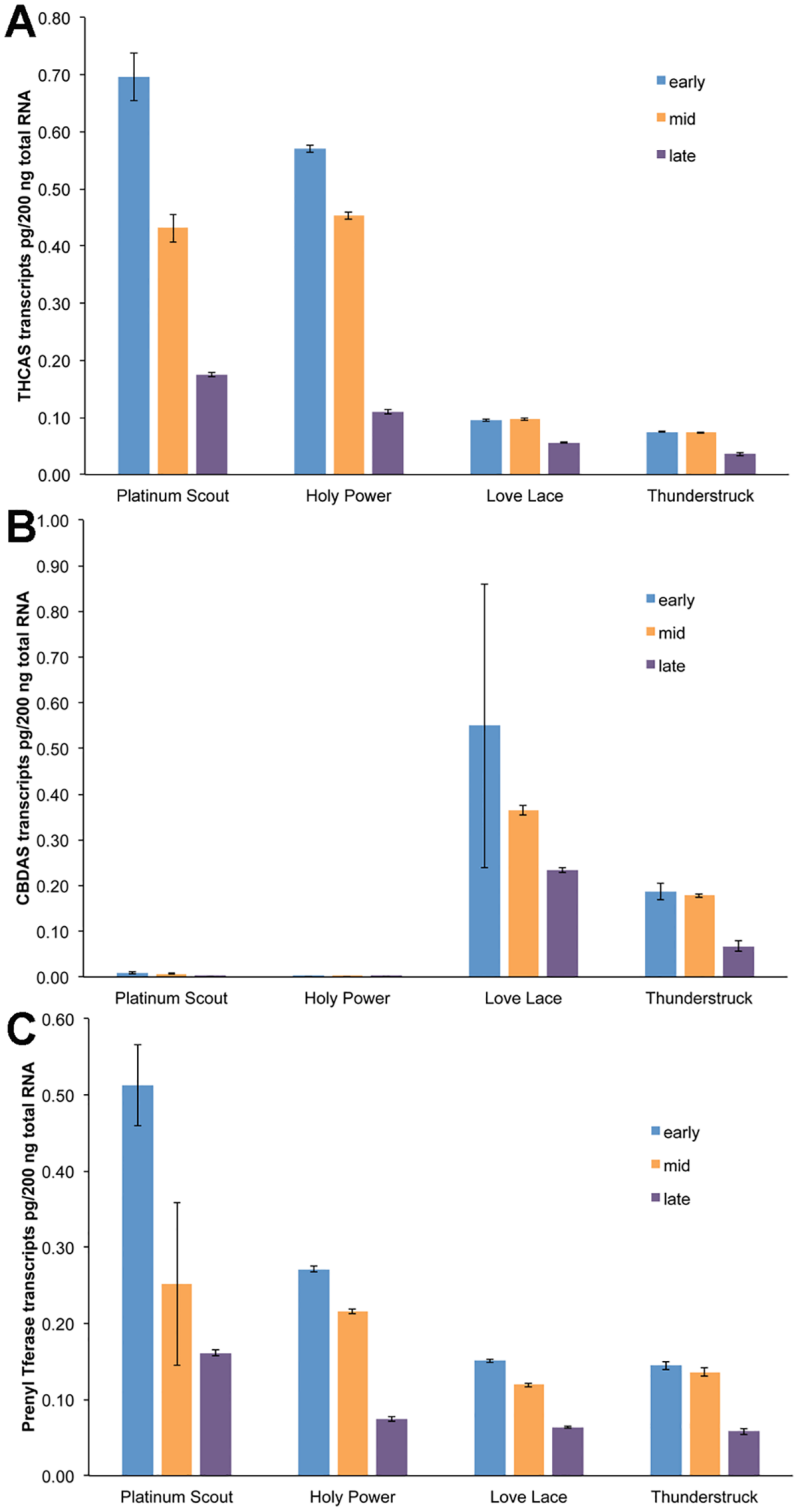
Transcript accumulation of cannabinoid biosynthetic genes during floral development. RNA was isolated from flowers collected from the indicated strains at early (blue), middle (orange) or late (purple) after the floral induction phase. Triplicate RNA isolations were assayed by qRT-PCR, using primers for THCAS (**A**), CBDAS (**B**) or prenyl transferase (**C**).

The transcript abundance of three cannabinoid biosynthetic genes, THCAS, CBDAS, and prenyl transferase was determined using qRT-PCR. The levels of these transcripts were determined in four strains, two producing high Δ9-THC levels in flowers (Platinum Scout and Holy Power) and two producing high CBD levels in their flowers (Love Lace and Thunderstruck). Plant samples for these studies were collected from greenhouse grown plants. Transcript levels for these three genes were quantified in leaves (Fig 7) and three different development stages in floral tissues (Fig 8A–8C).

Transcripts for all three genes were detected in leaves of all four strains. The level of CBDAS was the lowest of the transcripts in all four strains. The levels of transcripts for prenyl transferase and THCA synthase were similar within a strain. The two strains that accumulate CBD, Love Lace and Thunderstruck, had similar patterns of expression of all three genes, and the level of CBD synthase was higher in these two strains than in the strains that are higher in Δ9-THC. Platinum Scout, a high Δ9-THC strain, had the highest transcript levels for THCAS and prenyl transferase among the four strains. The Δ9-THC levels in the leaves of Platinum Scout are the highest of the four strains tested in this assay (Table 4).

Transcripts for all three genes were detected in the flowers of all four strains with notable variations. Maximal accumulation of a transcript occurred early in floral development and transcript levels decreased over time. This pattern was observed in all four strains for THCAS and for prenyl transferase (Fig 8A and 8C). Levels of CBDAS were also observed to follow this same developmental pattern in the two high CBD strains, Love Lace and Thunderstruck, while the levels of transcripts for CBDAS in Platinum Scout and Holy Power were barely detected (Fig 8B). The absolute levels of transcripts in leaves for these enzymes were 10 to 100-fold lower than in the floral RNA samples. This reflects the much greater abundance of the cannabinoids in the floral samples versus the leaf samples (Tables 3 and 4).

These results suggest that transcriptional regulation of the cannabinoid biosynthetic genes plays a role in the accumulation of Δ9-THC and CBD in the inflorescences of these plants. Transcript levels were higher in floral tissues than leaf tissues, and transcript accumulation of THCAS was much higher in lines that accumulate higher levels of Δ9-THC, and a similar relationship for CBDAS. Also, transcripts accumulated early in floral development with decreased transcript abundance as the flower matures, suggests that the enzymes translated from these transcripts persist in the flowers and continue to support cannabinoid biosynthesis. The genes and enzymes for capsaicinoid biosynthesis in fruit of *Capsicum sp*. follow a similar pattern, transcription peaks of genes for biosynthetic enzymes precede the metabolite accumulation [35, 41, 42,].

A few other groups have investigated the transcription of cannabinoid biosynthetic genes using qRT-PCR approaches, however in all of those cases, the levels of transcript are reported in relative terms not absolute amounts as we do here. This method difference may be the basis for some of the different results reported by these groups. THCAS and CBDAS levels in trichomes in flowers were described as stable during the latter stages of floral development in the *C*. *sativa* strain Bediol [43]. These authors also reported higher levels of THCAS and CBDAS in leaves versus flowers or flower trichomes, which is quite the inverse of the results reported here for four separate strains of *Cannabis* (Figs 7 and 8). The increase in CBDAS transcripts in flowers of hemp strains relative to THCAS transcripts in flowers of marijuana strains has been reported in a transcriptomic study [18] and in a gene mapping study [19]. A comparison of THCAS and CBDAS transcript levels among a number of strains that differ in the accumulation of Δ9-THC and CBD, revealed no association between final metabolite levels and transcript abundances in floral RNA [17]. Again we report here a positive relationship between transcript levels for THCAS or CBDAS and relevant metabolite accumulation. The difference in this case may again be due to the quantification of transcripts, as we determine absolute mass amounts and other groups report relative expression that has been normalized by a range of factors.

In summary we report a simple, robust and reliable method for the chemical characterization of two classes of bioactive compounds in medical *Cannabis* samples, terpenoids and cannabinoids. Given the myriad of health conditions treated by medical marijuana, detailed and complete compositional analyses are essential to determine the efficacy of this material for those conditions, and the optimal strain for specific conditions. The opportunity to predict high CBD floral samples from analyses on vegetative leaves should be explored further as a way to help producers increase the availability of this medically important cannabinoid.

## Supporting information

S1 Fig Accumulation of Δ9-THC in flowers following floral induction. Δ9-THC levels in samples collected from A. Bodhi Tree, B. Sour Willie, C. Cold Creek Kush, or D. Blue Dream at the days post-induction are represented. Samples are shown as flowers collected from the upper third of the plant (blue), middle third of the plant (red) or bottom third of the plant (green).
(TIF)

S2 Fig Paired comparisons of Δ9-THC levels in leaf and floral samples of *Cannabis* plants. Flowers (upper portion of the plant) and leaves (prior to floral induction) were collected from 21 *Cannabis* strains. The average floral content of each strain (n = 3–9) and the average leaf content (n = 3–9) are plotted.
(TIF)

S1 Table Cannabinoid and terpenoid composition in flower samples as cured commercially available medicinal marijuana. The terpenoid and cannabinoid abundances are reported as % dry wt for 72 strains, listed alphabetically by strain name, rows 2–73. The relative abundances of the terpenoids (% of total terpenoid) of these same samples are presented in rows 78–168. The results are organized by terpenoid type detected following the hierarchical clustering. Representative pie charts for the terpenoid composition are provided for each of the four terpenoid types: pinene, myrcene, terpinolene, and caryophyllene-limonene. A key to the color code for the pie chart is provided.
(XLSX)
