# Supplementary figures and images for: Accumulation of bioactive metabolites in cultivated medical Cannabis
Source: PLoS One. 2018 Jul 23;13(7):e0201119. doi: 10.1371/journal.pone.0201119 (PMC6056047; doi:10.1371/journal.pone.0201119)

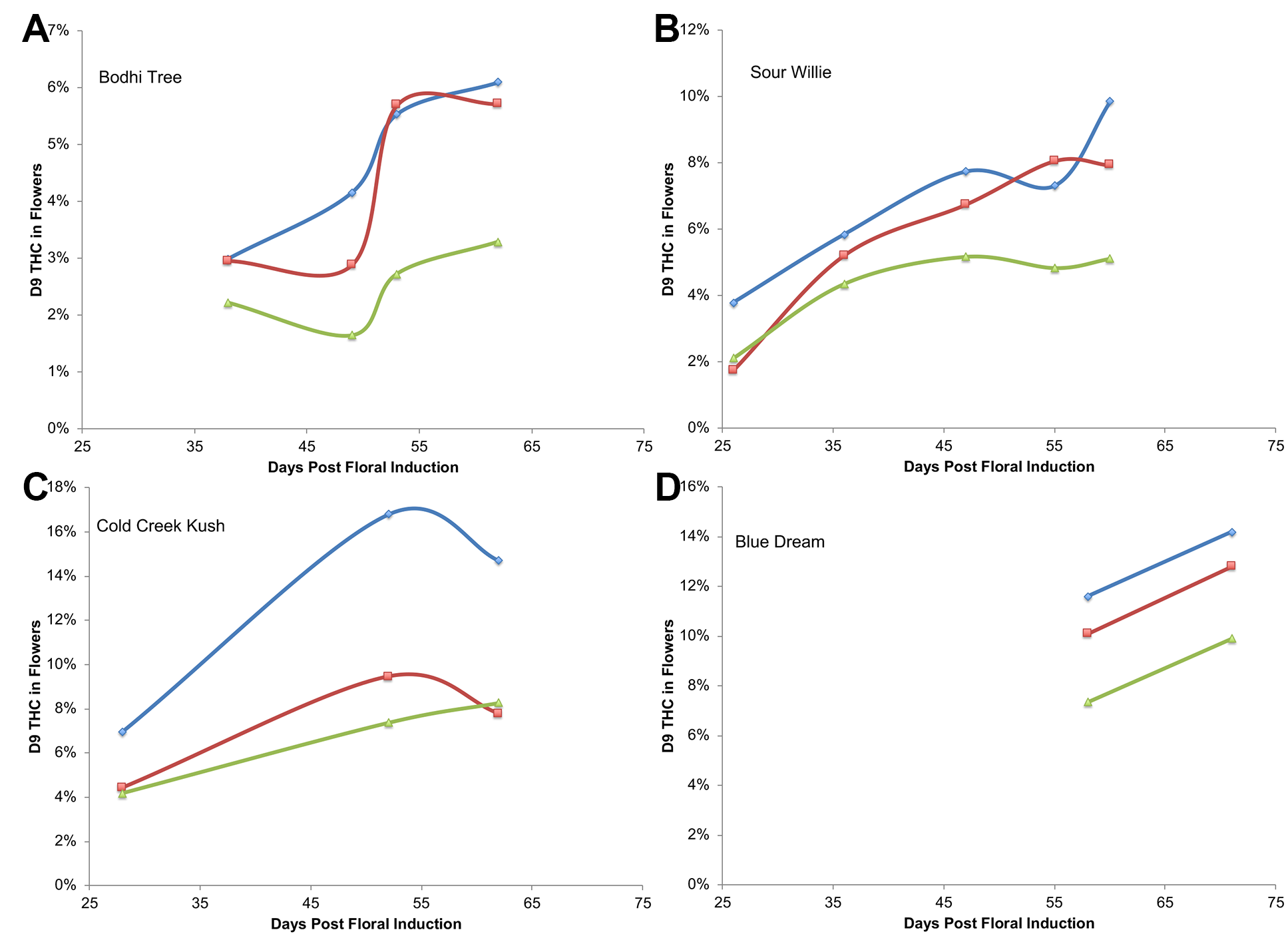

Supplement: S1 Fig — Δ9-THC levels in samples collected from A. Bodhi Tree, B. Sour Willie, C. Cold Creek Kush, or D. Blue Dream at the days post-induction are represented. Samples are shown as flowers collected from the upper third of the plant (blue), middle third of the plant (red) or bottom third of the plant (green). (TIF) [file pone.0201119.s001.tif]

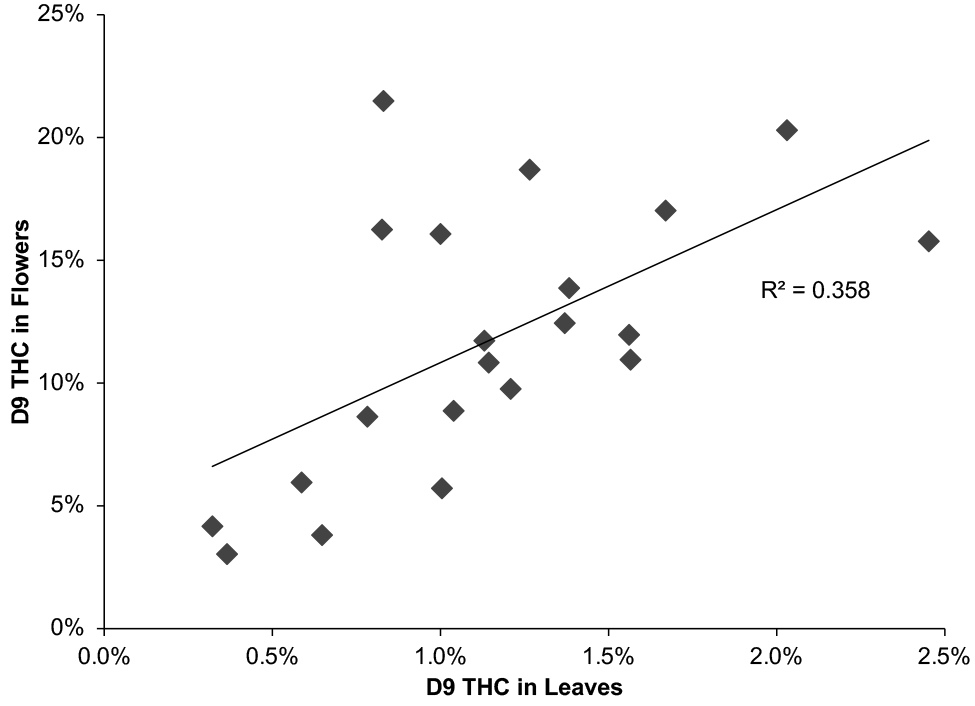

Supplement: S2 Fig — Flowers (upper portion of the plant) and leaves (prior to floral induction) were collected from 21 Cannabis strains. The average floral content of each strain (n = 3–9) and the average leaf content (n = 3–9) are plotted. (TIF) [file pone.0201119.s002.tif]
